# NOTUM Enhances Cartilage Repair via Wnt/β-Catenin Modulation in a Rabbit Osteochondral Defect Model

**DOI:** 10.3390/ijms27020647

**Published:** 2026-01-08

**Authors:** María López-Ramos, Gabriel Ciller, Cruz Rodríguez-Bobada, Patricia Quesada, Irene González-Guede, Ulises Gómez-Pinedo, Lydia Abasolo, Fernando Marco, Benjamín Fernández-Gutiérrez

**Affiliations:** 1 UGC of Rheumatology, Hospital Clínico San Carlos, Instituto de Investigación Sanitaria San Carlos, 28040 Madrid, Spain; maria.lopez.ramos@salud.madrid.org (M.L.-R.); igguede@salud.madrid.org (I.G.-G.); lydia.abasolo@salud.madrid.org (L.A.); 2 UGC of Traumatology, Hospital Clínico San Carlos, Instituto de Investigación Sanitaria San Carlos, 28040 Madrid, Spain; gabriel.ciller@salud.madrid.org (G.C.); fernando.marco@salud.madrid.org (F.M.); 3 Experimental Surgery Unit, Hospital Clínico San Carlos, Instituto de Investigación Sanitaria San Carlos, 28040 Madrid, Spain; mrbobadagonzalezcampo@salud.madrid.org (C.R.-B.); patricia.quesada@salud.madrid.org (P.Q.); 4 Laboratory of Neurobiology and Advanced Therapy, Instituto de Investigación Sanitaria San Carlos, 28040 Madrid, Spain; ulisesalfonso.gomez@salud.madrid.org

**Keywords:** osteoarthritis, cartilage repair, Wnt/β-catenin pathway, biomarkers, animal model

## Abstract

Osteoarthritis (OA) is the most common multifactorial joint disease characterized by progressive cartilage degradation and impaired tissue repair. Osteochondral defects represent a major clinical challenge within OA, as damage to cartilage and underlying bone can initiate degenerative changes and contribute to joint deterioration. The Wnt/β-catenin signaling pathway plays an important role in OA pathogenesis, and its dysregulation contributes to chondrocyte catabolism and cartilage loss. NOTUM, an extracellular Wnt inhibitor, has emerged as a potential therapeutic modulator capable of restoring signaling balance and promoting cartilage homeostasis. This study aimed to evaluate the efficacy of NOTUM compared with hyaluronic acid (HA), human adipose-derived mesenchymal stromal cells (hAd-MSCs), and Colchicine in a rabbit osteochondral defect model relevant to osteoarthritis. Twenty-seven New Zealand White rabbits underwent standardized femoral condyle injury and received single-dose treatments. Serum levels of cartilage biomarkers—Procollagen Type IIA N-terminal Propeptide (PIIANP) and Cartilage Oligomeric Matrix Protein (COMP)—were measured by ELISA at 4, 6, and 8 weeks post-surgery, and histological repair at week 12 was assessed using the modified O’Driscoll scoring system. NOTUM treatment significantly increased PIIANP and decreased COMP levels compared with HA, indicating enhanced cartilage synthesis and reduced degradation. Histological scores confirmed superior surface morphology and tissue composition in NOTUM-treated joints. These findings suggest that NOTUM performs a protective and regenerative effect through Wnt/β-catenin modulation, supporting the conclusion that it enhances osteochondral defect repair and motivating further studies of NOTUM as an OA therapy.

## 1. Introduction

Osteoarthritis (OA), the most common joint disease, is currently considered as a condition affecting all joint tissues, including cartilage, bone, synovium, and ligaments [1]. OA is a disabling condition with increasing incidence and prevalence every year. More than 500 million people are affected by osteoarthritis worldwide, suffering from pain and lack of physical activity between others [2]. As the most common orthopaedic condition, it is associated with a high health burden and implications not only for affected patients but also for healthcare systems [3]. Among all types, knee osteoarthritis (KOA) accounts for approximately 60.8% of the global disability burden associated with OA [4].

Osteochondral lesions represent a significant clinical challenge due to the limited intrinsic regenerative capacity of articular cartilage [5,6,7]. These lesions constitute a demanding condition in orthopaedic practice and are associated with a substantial socioeconomic burden, particularly in an ageing population. It has been observed that there is cross-talk or chemical communication via cytokines between these tissues, stimulating each other and giving rise to inflammatory peptides [8]. Osteochondral defects may arise following traumatic injury in an otherwise healthy joint and, if left untreated or inadequately repaired, are expected to trigger progressive joint deterioration and ultimately contribute to the development of osteoarthritis. When chronic, this multifactorial degenerative disorder results in cartilage degeneration, subchondral bone exposure, and periarticular soft tissue changes [9].

Despite numerous therapeutic options aimed at symptom relief, current treatments for osteoarthritis fail on reversing disease progression, and no pharmacological interventions have consistently demonstrated structural improvement of the joint. This highlights the urgent need to explore novel molecular targets capable of modifying disease progression.

Animal models are essential for preclinical evaluation of osteochondral repair strategies, providing controlled environments to assess tissue response and treatment efficacy [10,11]. Among these, the New Zealand White rabbit model has been widely employed due to its appropriate joint size, manageable handling, and biological response [12,13].

Recent advances in biological therapies have focused on enhancing joint homeostasis and modulating inflammation to support tissue regeneration; but, despite this, there is still no clear evidence confirming their clinical effectiveness. Intra-articular hyaluronic acid (HA) injections have demonstrated to improve joint function, relieve pain, and reduce the dosage of analgesics [14]. Human adipose tissue-derived mesenchymal stromal cells (hAd-MSCs) possess self-renew and multidirectional-differentiation potentials and can modulate immunity, resist inflammation, promote angiogenesis, and enhance tissue regeneration [15,16]. Further, Colchicine could modulate innate immune responses by interacting with toll-like receptor 7 but, so far, evidence of effectiveness are contradictory [17,18]. Colchicine is a well-known anti-inflammatory treatment used in gout, a process that shares common inflammatory pathways with osteoarthritis. Furthermore, it is important to emphasize that Colchicine is also used as a treatment for auto-inflammatory diseases [19,20].Although previous studies on colchicine in OA are limited and show inconclusive results [21], we included it as a comparator because it represents a clinically available anti-inflammatory agent currently under investigation for joint disorders [22,23]. Its inclusion allows us to evaluate NOTUM’s regenerative potential relative to an agent of clinical relevance, focusing on osteochondral repair outcomes rather than mechanistic equivalence.

Additionally, pharmacological agents such as signalling pathway modulators are being studied as promising candidates for regulating the inflammatory microenvironment and promoting cartilage repair. One of the pathways involved in the pathophysiology of OA is Wnt/b-catenine pathway which has been seen to be overexpressed in patients [24,25,26]. NOTUM, a negative regulator of Wnt signaling, has been proposed as a potential therapeutic candidate for OA [27,28,29].

In this context, the evaluation of biochemical markers of cartilage metabolism provides a valuable complement to structural and histological assessments in experimental OA models. As OA progression results from the imbalance between anabolic and catabolic activities within the joint microenvironment, serum biomarkers can reflect these dynamic processes non-invasively [30,31]. Among them, Procollagen Type IIA N-terminal propeptide (PIIANP) is indicative of type II collagen synthesis and cartilage matrix formation, whereas Cartilage Oligomeric Matrix Protein (COMP) reflects extracellular matrix degradation and chondrocyte stress [32,33]. Monitoring these biomarkers allows for a longitudinal understanding of how each therapeutic strategy—such as HA, hAd-MSCs, colchicine, or NOTUM—modulates cartilage turnover and joint homeostasis. Therefore, their integration into preclinical studies not only enhances the assessment of osteochondral repair but also strengthens the translational bridge between experimental findings and clinical outcomes in osteoarthritis research.

Therefore, the aim of this study was to compare the effects of different therapeutic approaches—including HA, hAd-MSCs, Colchicine, and NOTUM—in rabbit model of osteochondral defect, a condition closely related to the development of osteoarthritis. Treatment outcomes were evaluated through histological analysis of the femoral condyles and longitudinal monitoring of serum PIIANP and COMP levels, to determine their utility as biochemical indicators of osteochondral repair and to support the translational relevance of this experimental model.

## 2. Results

### 2.1. Comparative Analysis of Serum COMP and PIIANP Levels

**Figure 1:**
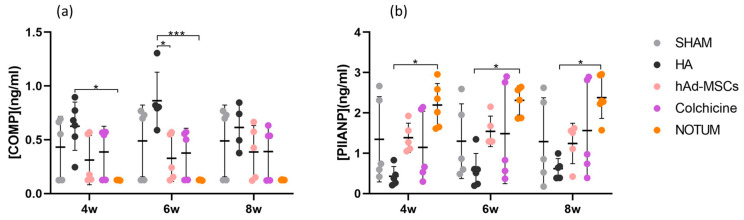
Relative levels of (**a**) COMP and (**b**) PIIANP in serum of blood peripheral samples in the SHAM group (*n* = 5), HA group (*n* = 6), hAd-MSCs group (*n* = 5), Colchicine group (*n* = 5) and NOTUM group (*n* = 6) taken at week 4 (4w), week 6 (6w) and week 8 (8w) post-surgery. Statistically significant differences between groups are indicated: *, *** (*p* < 0.05, *p* < 0.0001, respectively). SHAM, Control; HA, Hyaluronic Acid; hAd-MSCs, Human Adipose Tissue Mesenchymal Stromal Cells.

COMP and PIIANP serum levels of peripheral blood were measured by ELISA at weeks 4, 6 and 8 post-surgery. COMP levels were significantly higher in the HA group compared to the NOTUM group at weeks 4 and 6 (*p* = 0.0448 95%CI = [0.01, 0.99], *p* = 0.0004 95%CI = [0.22, 1.25], respectively) and compared to the hAd-MSCs group at week 6 (*p* = 0.0363) 95%CI= [0.02, 1.05] (Figure 1a). PIIANP levels were significantly higher in the NOTUM group than in the HA group at all-time points (week 4: *p* = 0.0140 95%CI = [−3.34, −0.20]; week 6: *p* = 0.0100 95%CI = [−3.23, −0.23]; week 8: *p* = 0.0145 95%CI = [−3.33, −0.19]) (Figure 1b).

### 2.2. Histological Assessment of Osteochondral Repair

**Figure 2:**
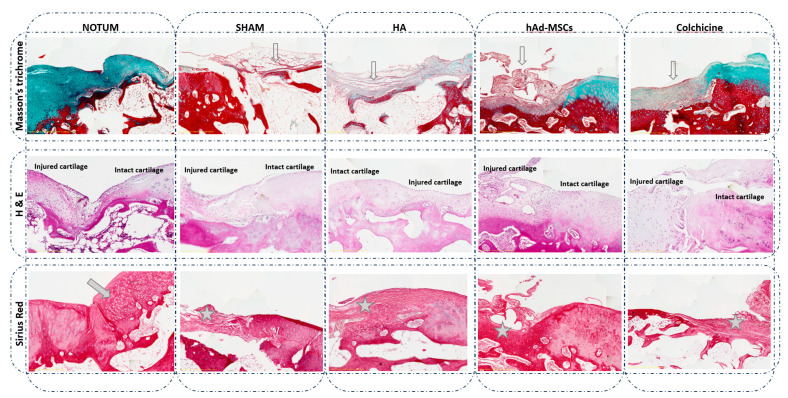
Representative histological knee osteochondral sections stained with Masson’s trichrome, Haematoxylin and Eosin (H&E), and Sirius Red. Comparison between treatments: NOTUM, SHAM, HA, hAd-MSCs and Colchicine. Masson’s trichrome images show a loss of green/blue staining, indicating decreased collagen fibre content and reduced extracellular matrix organization in all groups compared to NOTUM. Surface disruption is also observed in SHAM, HA and hAd-MSCs histological sections (hollow arrow). H&E images display general tissue morphology, highlighting structural and cellular alterations between groups. Severe hypocellularity, hypercellularity or cluster formation is observed in all groups compared to NOTUM. Sirius Red images reveal the difference in the nature of the new tissue formed. Hyaline or similar cartilage is shown in NOTUM (arrow) while fibrous tissue is observed in SHAM, HA, hAd-MSCs and Colchicine groups (asterisk). SHAM, Control; HA, Hyaluronic Acid; hAd-MSCs, Human Adipose Tissue Mesenchymal Stromal Cells.

Histological evaluation of the femoral condyles was performed at week 12 using the modified O’Driscoll scoring system by two independent blinded observers. Table 1 shows the characteristics of the groups based on the modified O’Driscoll visual histological assessment score used. For Surface/Morphology, the NOTUM group showed a trend toward higher scores compared with SHAM group (*p* = 0.0556, 95% CI [−5.47, 0.07]) and significantly better results than the HA group (*p* = 0.0496, 95% CI [−5.50, −0.01]). For Composition and Tissue integration, NOTUM treatment group results were better than HA and Colchicine groups (*p* = 0.0230 95%CI = [−7.70, −0.63], *p* = 0.0340 95%CI = [−8.63, −0.38], respectively). Finally, the scores of NOTUM group in Cellular response showed a trend toward being higher (but non-significant) than Colchicine group results (*p* = 0.0544 95%CI = [−4.55, 0.05]). All these differences are illustrated in Figure 2.

### 2.3. Histological Damage Distribution Across Treatment Groups

Scores were categorized according to damage severity: 0 = no damage, 1 = mild, 2 = moderate, and 3 = severe.

**Figure 3:**
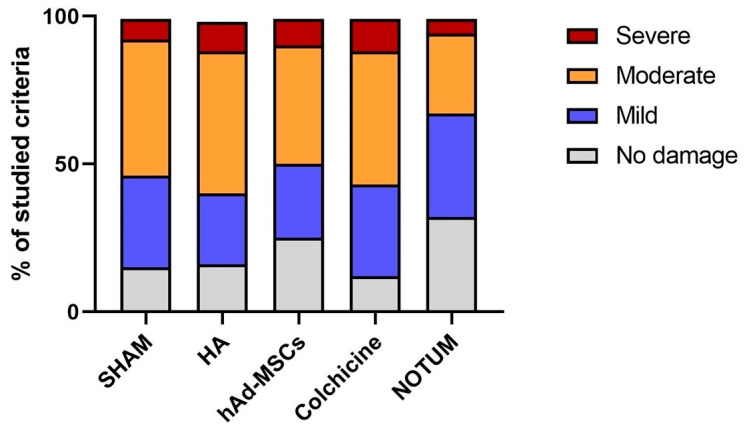
Distribution of histological damage categories across treatment groups. Stacked bar chart showing the relative distribution of knees classified into four categories of histological damage (0 = no damage, 1 = mild, 2 = moderate, 3 = severe) according to the modified O’Driscoll scoring system. Bars represent the percentage of knees in each category within each treatment group. SHAM, Control; HA, Hyaluronic Acid; hAd-MSCs, Human Adipose Tissue Mesenchymal Stromal Cells.

The distribution of histological damage severity differed significantly among the treatment groups (χ^2^ test, *p* < 0.0001). As illustrated in Figure 3, the NOTUM group showed the highest proportion of knees with no or only mild histological damage and the lowest proportion with severe damage compared to all other experimental groups. In contrast, SHAM, HA, hAd-MSCs, and Colchicine groups exhibited higher proportions of moderate and severe lesions. The stacked bar chart in Figure 3 displays the percentage distribution of damage categories across groups.

## 3. Discussion

This study highlights the therapeutic potential of NOTUM in osteochondral repair, demonstrating its ability to regulate cartilage metabolism and enhance tissue regeneration. Unlike conventional therapies that primarily target inflammation or symptoms, NOTUM directly targets the dysregulated Wnt/β-catenin pathway, known to be dysregulated in degenerative cartilage [29,34,35]. Moreover, recent studies have reported reduced NOTUM levels in patients with OA, suggesting that its decrease may contribute to pathological Wnt activation in this context [36]. By inhibiting excessive Wnt activity, NOTUM promotes a reparative microenvironment, as reflected by increased PIIANP and reduced COMP levels, corroborated by improved histological scores. These findings support NOTUM as a promising regenerative strategy for osteochondral repair, with strong translational relevance for conditions that may progress to osteoarthritis.

Interestingly, previous studies have identified NOTUM as a promising modulator of bone metabolism through its inhibitory action on the Wnt/β-catenin signaling pathway, which plays a central role in osteoblast differentiation and bone formation. In the context of osteoporosis, pharmacological inhibition of NOTUM has been explored as a strategy to enhance Wnt activity, thereby stimulating bone formation and improving bone mass and strength [37,38]. Conversely, in osteoarthritis, where excessive Wnt activation contributes to aberrant chondrocyte differentiation and cartilage degradation, the administration of exogenous NOTUM may restore the balance between anabolic and catabolic processes within the joint.

Our results demonstrated differential responses among treatment groups. Serum COMP levels, a marker of cartilage matrix degradation, were significantly higher in the HA group at early time points (weeks 4 and 6) compared to NOTUM, and at week 6 compared to hAd-MSCs. This suggests that HA treatment may be associated with a higher degree of cartilage turnover or matrix breakdown during the early phase of repair. This observation is consistent with previous studies reporting that HA, while effective in reducing joint inflammation and pain, may not directly inhibit matrix catabolism [39,40]. Conversely, NOTUM treatment was associated with significantly lower COMP levels, supporting its potential role in stabilizing cartilage matrix by downregulating catabolic signalling, likely via inhibition of the Wnt/β-catenin pathway [29,34].

Regarding cartilage anabolism, PIIANP levels—a marker of collagen type II synthesis—were significantly elevated in the NOTUM group compared to HA at all-time points (weeks 4, 6, and 8), indicating a sustained pro-regenerative response. These findings strength the hypothesis that modulation of Wnt signalling by NOTUM not only limits matrix degradation but also favours anabolic remodelling of cartilage [35]. Interestingly, hAd-MSCs, despite their known regenerative potential [15,16], did not show significant differences in PIIANP levels compared to the control groups, possibly due to variability in cell engraftment or survival, or to the immunomodulatory rather than pro-synthetic role of MSCs in this context.

Histological evaluation at week 12 using the modified O’Driscoll scoring system further supported the biochemical findings. The NOTUM-treated group showed in general improved tissue morphology, surface regularity, and cellular response, aligning with biomarker trends and suggesting superior structural repair. These findings align with the proposed mechanism of NOTUM as a modulator of the Wnt/β-catenin signalling pathway, which has been implicated in osteoarthritis pathophysiology and cartilage degeneration [27,29]. By regulating this pathway, NOTUM may reduce excessive catabolic activity and promote a microenvironment more favourable for cartilage preservation.

In contrast, while HA has been widely reported to improve joint homeostasis and modulate inflammation, their effects in this study appeared less pronounced in terms of reducing severe cartilage damage [41,42]. This could be attributed to differences in mode of action, timing, or dosing, as well as potential limitations of the single-injection protocol employed. Although the group treated with hAd-MSCs did not reach statistical significance in contrast with previous studies, its data exhibit a trend toward improvement is evident, which might be further enhanced by administering higher treatment doses. Similarly, Colchicine, administered orally, did not demonstrate a comparable protective effect, which is consistent with previous reports of its inconsistent efficacy in OA [17,22,43,44]. These data could be due to a study conducted over a very long period of time, during which the anti-inflammatory effects may have already been lost. For this reason, it would be advisable to carry out further studies on Colchicine treatment, either with a sample collection period earlier than the current one or, alternatively, through continuous administration of doses that allow the effect to be maintained over time.

Importantly, the combination of 12-week histological data and 8-week biochemical follow-up provided a multidimensional assessment of cartilage repair. The correlation between decreased COMP levels and improved histological features highlights the relevance of NOTUM biomarker in reflecting treatment response. Likewise, PIIANP increases paralleled structural recovery, especially in the NOTUM group, validating its role as a surrogate marker for cartilage synthesis. Although the selected concentration has been justified, it remains possible that different dosing levels could modulate Wnt/β-catenin signaling with varying magnitudes, revealing threshold or saturation effects not captured here. Future studies including structured dose–response analyses will be essential to identify the optimal therapeutic window and further refine the translational potential of NOTUM in osteochondral repair.

This study has some limitations. We acknowledge that small and slightly unequal group sizes may reduce statistical power and increase variability; however, using both knees per animal maximized observations while minimizing total animal use. The biochemical follow-up period was limited to 8 weeks, which, although sufficient to capture early cartilage repair, does not allow assessment of long-term durability and tissue maturation. The creation of a large full-thickness defect in the medial condyle can generate an inflammatory environment affecting the entire joint, including the lateral condyle. Therefore, while paired comparisons within the same joint allow for direct evaluation of treatment effects, the control tissue may not be entirely free from inflammatory influences. Histological scoring, despite being performed by two blinded observers, may be influenced by inter-observer variability. This limitation could be mitigated in future experiments by including a larger panel of evaluators. Although all groups received oral pineapple juice and an intra-articular saline injection to avoid vehicle-related bias, we acknowledge that these interventions could theoretically influence the joint microenvironment or systemic inflammatory responses. However, the single low-volume administration used in this study makes it unlikely that either pineapple juice or saline produced meaningful biological effects on cartilage repair. Importantly, these controls were uniformly applied across groups, minimizing their potential impact on comparative outcomes. Additionally, while the rabbit model is widely used and translationally relevant, it cannot fully replicate human osteoarthritis. It is important to note that this study focuses on osteochondral repair, a process that, if left untreated, can contribute to the development of osteoarthritis. Future studies should aim to evaluate NOTUM in animal models specifically validated for OA pathophysiology, allowing a direct comparison of its effects on established osteoarthritis versus osteochondral repair. Therefore, progressing toward an early-phase clinical trials will help validate the potential of NOTUM as a disease-modifying therapy for osteoarthritis.

Another key limitation of the present study is that the involvement of the Wnt/β-catenin pathway was inferred from the known biological activity of NOTUM and from the observed structural and biochemical outcomes, but it was not directly evaluated in joint tissues. We did not perform molecular or immunohistochemical analyses of pathway markers, which would have strengthened the mechanistic interpretation. Future work will include targeted analyses—such as immunohistochemistry, qPCR, or Western blotting—to validate the specific effects of NOTUM on Wnt signaling during cartilage repair.

Despite all these limitations, this study provides a comprehensive evaluation of osteochondral repair by combining biochemical and histological analysis. The inclusion of multiple treatment arms—including NOTUM, HA, MSCs, and Colchicine—allows direct comparison of therapeutic efficacy. The use of validated serum biomarkers (COMP and PIIANP) together with blinded histological scoring enhances the reliability and translational relevance of the findings. Moreover, this is the first preclinical investigation to evaluate intra-articular NOTUM administration as a modulator of Wnt/β-catenin signaling in a rabbit model of osteochondral defect, highlighting its potential to enhance cartilage regeneration.

## 4. Materials and Methods

### 4.1. Animal Model

**Figure 4:**
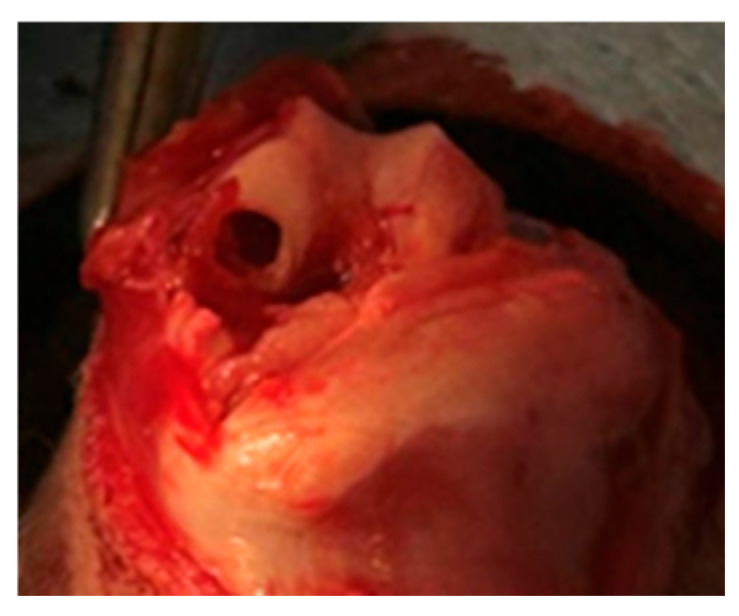
Osteochondral defect view of the medial femoral condyle of a rabbit knee.

Twenty-seven male New Zealand White rabbits (*Oryctolagus cuniculus*), 7–11 months old and weighing 3.9 ± 0.65 kg, underwent an anterior approach to both knees. An osteochondral lesion measuring 4 mm thick and 5 mm deep was performed on the medial femoral condyle under general anaesthesia (Figure 4). Analgesia (Buprenorphine) was provided until 72 h after surgery and all animals were immediately allowed to move freely.

The animal procedures were approved by the Ethics Committee on Animal Experimentation of the Hospital Clínico San Carlos (Madrid, Spain) and in accordance with the approved guidelines (PROEX 110.6/22), approved on 1 August 2021.

### 4.2. Intra-Articular Injection

One-week post-surgery, animals were randomly divided to one of the following treatment groups: (1) SHAM (*n* = 5), (2) Hyaluronic Acid (HA, *n* = 6), Human Adipose Tissue Mesenchymal Stromal Cells (hAd-MSCs, *n* = 5), Colchicine (*n* = 5) and NOTUM (*n* = 6). Single intra-articular injections were administered with animals in the supine position under isoflurane inhalational anaesthesia (1.5–2%) (Baxter, Deerfield, IL, USA). A 23-gauge needle was used to inject treatments into both knee joints via the patellar ligament. Both knees of each rabbit received the same treatment.

SHAM group: 1 mL saline solution (*n* = 5, 10 knees). HA group: 1 mL Hylan G-F 20 (8 mg, Synvisc, Genzyme, a Sanofi company, Cambridge, MA, USA) (*n* = 6, 12 knees). hAd-MSCs group: 1 mL containing 1 × 10^6^ human cells diluted in Hypothermosol (Stemcell Technologies, Vancouver, BC, Canada) (*n* = 5, 10 knees). Colchicine group: 1 mL oral pineapple juice containing 60 µg of Colchicine (Fisher Scientific, Waltham, MA, USA) (*n* = 5, 10 knees). NOTUM group: 1 mL saline solution containing 1.2 µg rh-NOTUM (Biotechne, Minneapolis, MN, USA) (*n* = 6, 12 knees).

The hAd-MSCs were provided by the Cell Therapy Unit (Clean Room) of Hospital Clínico San Carlos. They were obtained from lipoaspirates of healthy adult donors between 18 and 45 years of age, after signing the corresponding informed consent and in compliance with RD 9/2014. This part of the study was conducted in accordance with the Declaration of Helsinki, and approved by Research Ethics Committee of the IdISSC-HCSC (C.I. 21/133-E) in accordance with Spanish regulation (RD 1090/2015) approved on 12 July 2021. The passage used for the experiments was passage 3.

For Colchicine, the dose (60 µg in 1 mL pineapple juice, orally) was calculated from the standard human adult dose (approximately 1 mg for a 70 kg adult), adjusted for rabbit body weight and blood volume.

For NOTUM, the chosen dose (1.2 µg per knee) was determined from in vitro experiments in rabbit BM-MSCs, identifying the minimal concentration required to achieve a measurable reduction in Wnt3A expression, and thereby modulate Wnt/β-catenin signaling. Additionally, the selected dose was informed by concentrations used in preclinical studies of Lorecivivint, another small-molecule modulator of the Wnt pathway, which provided a relevant biological reference [45,46].

All treatments were administered via intra-articular injection except colchicine, which was given orally, following normal clinical practice. The different administration route was intentionally maintained because Colchicine is not used intra-articularly in humans, allowing us to evaluate its potential effect on osteochondral repair under conditions that mimic real-world therapeutic use. The Colchicine group received a single oral dose of 60 µg of lyophilized Colchicine dissolved in 1 mL of pineapple juice. Pineapple juice was chosen to serve as a swallowing vehicle and because of the properties of the enzyme bromelain, which is capable of digesting hairballs in the animals’ stomachs.

Since the other experimental groups received an intra-articular injection, in order to avoid potential bias arising solely from the puncture procedure, the animals in Colchicine group were given an intra-articular injection of 1 mL of 0.9% saline solution under the same aseptic conditions and technique used for the other treatments.

Similarly, to avoid any bias caused by the oral administration of pineapple juice to Colchicine group, all animals in the other groups ingested 1 mL of pineapple juice on the same day as the intra-articular treatment. Detailed administration protocols are summarized in Table 2.

### 4.3. Sample Collection

At weeks 4, 6 and 8 post-surgery; 3 mL of blood were collected from the central auricular artery using serum collection tubes (Becton Dickinson, Franklin Lakes, NJ, USA). Local anaesthetic cream with lidocaine (Emla, Aspen, uMhlanga, South Africa) was applied in the ear to minimize discomfort. Blood samples were centrifuged at 3000 rpm for 15 min, and serum was stored at −80 °C until analysed.

### 4.4. Analysis of Serum Protein Expression by ELISA

Quantitative COMP and PIIANP levels in serum were measured using a commercial sandwich enzyme-linked immunosorbent assay (ELISA) kits (Mybiosource, San Diego, CA, USA), according to the manufacturer’s instructions. Rabbit Procollagen Type IIA N-Prop ELISA, #MBS9399068 with a detection range of 0.156–10 ng/mL was the one used for PIIANP protein. Rabbit cartilage oligomeric matrix protein ELISA, #MBS2600996 with a detection range of 3.12–100 ng/mL was the one used for COMP.

### 4.5. Tissue Sampling and Preparation

Three months after surgery, euthanasia was performed by intravenous injection of sodium thiopental (Braun, Melsungen, Germany) and propofol (Baxter, USA), to avoid unnecessary pain and suffering.

After euthanasia, both femoral condyles were removed through careful dissection, avoiding tissue damage. Samples were fixed in 4% formaldehyde stabilized at pH 7 (Casa Álvarez, Madrid, Spain) for 4 days at 4 degrees. Then, they were decalcified for 7–10 days, depending on the sample’s needs, using Decalcifier II (Leica, Wetzlar, Germany). Once the desired decalcification was achieved, the condyles were prepared and sectioned along their midline into two blocks (damaged area or internal condyle and control area or external condyle). The samples were embedded in paraffin using a paraffin embedding device (Leica). Finally, they were cut using a conventional manual microtome RM2235 (Leica) into 5-μm-thick sections. Sections were then dewaxed in xylene and hydrated through graded ethanol series before being stained with Hematoxylin and Eosin (H&E), Safranin-O/Fast Green, Picro Sirius Red and Masson’s Trichrome. All staining processes were performed at room temperature.

### 4.6. Histological Evaluation of the Knee Joints

One histologic section from each stain and site was evaluated using a modified O’Driscoll scoring system [47,48] (Table 3) by two blinded, experienced researchers using an optic microscope Leica DMI 4000 B (Leica). For histological evaluation, external femoral condyles of each knee were designated as the control specimen. The score assessed tissue composition, surface morphology, cellular response, and subchondral bone repair.

### 4.7. Statistical Analysis

Data were expressed as mean ± standard deviation (x ± s). Comparisons between two groups used unpaired *t*-tests (parametric) or Mann–Whitney tests (non-parametric). Comparisons among three or more groups were analysed with two-way ANOVA followed by Tukey’s post hoc correction for multiple comparisons. Categorical histological scores (0–3) were organized into contingency tables and compared using the Chi-square (χ^2^) test or Fisher’s exact test when appropriate. Outliers were detected using the ROUT method (Q = 1%). Statistical significance was set at *p* < 0.05 and represented with ± 95% confidence interval (95%CI) when possible. Analyses were performed using GraphPad Prism 8 (GraphPad Software, San Diego, CA, USA).

The sample size for this study was determined based on previous work from the Orthopedic Surgery and Traumatology research group at Hospital Clínico San Carlos, which employed the same rabbit osteoarthritis model [49,50]. The calculation also adhered to the “3Rs” principle (Replacement, Reduction, and Refinement) for animal research. Although determining sample sizes for in vivo experiments and their subanalyses is inherently complex, we estimated that 10–12 samples per group would be sufficient to detect a 6-point difference in histological evaluation scores (ICRS) with 80% statistical power. To minimize the number of animals used, osteochondral defects were created in both knees of each rabbit.

## 5. Conclusions

In conclusion, this study provides preliminary evidence that NOTUM treatment may improve osteochondral repair in a rabbit model of osteochondral defect, a condition known to predispose to osteoarthritis. These findings support further preclinical investigation and provide a foundation for potential translational applications in the management of osteochondral lesions. In addition, the use of serum biomarkers such as COMP and PIIANP provides a valuable, non-invasive method for monitoring treatment efficacy, and supports their integration into future preclinical and clinical studies on OA therapies which may constitute the initial steps in improving the management of these patients.

## Figures and Tables

**Table 1:** Histological assessment score results of studied groups.

	Surface/Morphology				
	SHAM	HA	hAd-MSCs	Colchicine	NOTUM
Mean ± SD					
	8.2 ± 2.90	8.25 ± 3.22	6.90 ± 4.10	7.90 ± 3.14	5.50 ± 3.26
*p*-value					
SHAM		0.9701	0.4232	0.8269	0.0556
HA			0.3968	0.8002	**0.0496**
hAd-MSCs				0.5478	0.3823
Colchicine					0.0960
	**Composition/Tissue Integration**				
	**SHAM**	**HA**	**hAd-MSCs**	**Colchicine**	**NOTUM**
Mean ± SD					
	12.40 ± 3.06	13.17 ± 1.95	11.10 ± 5.82	13.50 ± 3.06	9.00 ± 5.58
*p*-value					
SHAM		0.4840	0.5398	0.4325	0.1009
HA			0.2599	0.7598	**0.0230**
hAd-MSCs				0.2637	0.3987
Colchicine					**0.0340**
	**Cellular Response**				
	**SHAM**	**HA**	**hAd-MSCs**	**Colchicine**	**NOTUM**
Mean ± SD					
	5.70 ± 2.06	6.33 ± 2.74	6.30 ± 3.56	7.00 ± 2.21	4.75 ± 2.83
*p*-value					
SHAM		0.5540	0.6500	0.1903	0.3879
HA			0.9804	0.5431	0.1780
hAd-MSCs				0.6038	0.2685
Colchicine					0.0544
	**Subchondral Bone**				
	**SHAM**	**HA**	**hAd-MSCs**	**Colchicine**	**NOTUM**
Mean ± SD					
	2.80 ± 0.79	2.67 ± 1.16	2.50 ± 1.43	2.50 ± 1.08	1.83 ± 1.59
*p*-value					
SHAM		0.9757	0.6099	0.3645	0.1971
HA			0.7847	0.5184	0.2300
hAd-MSCs				>0.9999	0.3433
Colchicine					0.3772

Significant *p*-values (*p* < 0.05) are highlighted in bold. SHAM (*n* = 10), HA (*n* = 12), hAd-MSCs (*n* = 10), Colchicine (*n* = 10), NOTUM (*n* = 12). SHAM, Control; HA, Hyaluronic Acid; hAd-MSCs, Human Adipose Tissue Mesenchymal Stromal Cells.

**Table 2:** Administration groups for the treatments against OA.

Group	Administration	Amount	Treatment
SHAM	Intra-articular	1 mL	Saline Solution
HA	Intra-articular	1 mL (8 mg)	Hylan G-F20
hAd-MSCs	Intra-articular	1 mL (1 × 10^6^ cells)	Ad-MSCs
Colchicine	Oral	1 mL (60 µg)	Colchicine 97%
NOTUM	Intra-articular	1 mL (1.2 µg)	rh-NOTUM

SHAM, control group; HA, hyaluronic acid; hAd-MSCs, human adipose tissue-derived mesenchymal stromal cells; rh, recombinant human. SHAM (*n* = 10), HA (*n* = 12), hAd-MSCs (*n* = 10), Colchicine (*n* = 10) and NOTUM (*n* = 12).

**Table 3:** Criteria (grading) for histologic evaluation. The modified O’Driscoll score.

Characteristic		Grade
Composition/Tissue integration		
Nature of the predominant tissue	Hyaline or similar cartilage	0
	Poorly identifiable cartilage	1
	Fibrous tissue	2
% Hyaline cartilage in the damaged area	>75%	0
	>50%	1
	>25%	2
	<25%	3
Bonding of the new tissue to the adjacent cartilage	Bonding at both ends of graft	0
	One/Partial both	1
	No bonded	2
Bonding of repair cartilage to the new subchondral bone	Complete	0
	>50%	1
	<50%	2
Surface/Morphology		
Surface structure	Smooth-intact	0
	Fissures	1
	Disruption	2
Integrity of the new area	Normal	0
	Slight disruption	1
	Severe disruption	2
Thickness of new tissue vs. adjacent tissue	Normal	0
	Increased	1
	Decreased	2
Cellular response		
Cellular changes in cartilage injury	Normal staining, no clusters	0
	Moderate staining, mild clusters	1
	Slight staining, moderate hypocellularity	2
	Poor staining, severe hypocellularity	3
Cellular changes in adjacent cartilage	Normal staining, no clusters	0
	Moderate staining, mild clusters	1
	Slight staining, moderate hypocellularity	2
	Poor staining, severe hypocellularity	3
Subchondral bone		
Subchondral bone reconstruction	Complete	0
	>50%	1
	<50%	2
		Max 23

## Data Availability

The original contributions presented in this study are included in the article. Further inquiries can be directed to the corresponding author.

## Abbreviations

The following abbreviations are used in this manuscript:

COMP	Cartilage Oligomeric Matrix Protein
HA	Hyaluronic Acid
H&E	Hematoxylin and Eosin
hAd-MSCs	Human Adipose-derived Mesenchymal Stromal Cells
KOA	Knee Osteoarthritis
OA	Osteoarthritis
PIIANP	Procollagen Type IIA N-terminal Propeptide
